# Attitudes to apps for continence

**Published:** 2012-06-15

**Authors:** Kate Stephen, Gaener Rodger, Grant Cumming

**Affiliations:** University of the Highlands and Islands, UK; University of the Highlands and Islands, UK; University of the Highlands and Islands, UK

**Keywords:** apps, continence, adherence, exercise, attitudes

## Abstract

**Background:**

Urinary incontinence (UI) is a common condition in women which can be caused by the weakening or dysfunction of pelvic floor muscles as a result of ageing, pregnancy or obesity. Prevalence of UI is higher in women than that of diabetes, depression and hypertension but rates are difficult to establish because there is evidence of a reluctance to seek help and assumptions that symptoms are normal and untreatable. Women can become increasingly depressed and isolated. The first line of treatment for UI is pelvic floor muscle training (PFMT) and effectiveness has been shown in over 50 randomised controlled trials. However, there are gaps in research about motivation and adherence to exercise. Research about mobile participatory healthcare has identified the role of technology in addressing problems with adherence. There has been an increase in the use of smart phones to access the internet and in the number of health related applications but there is a lack of research about how individuals interact with this new technology and a lack of understanding about effectiveness of different elements of the technology. Mobile phone apps have been developed to support women in PFMT. The approaches used to promote exercise include; improving incontinence symptoms, strengthening muscles after pregnancy and increasing sexual sensation. The apps have features such as; reminders, counts and graphs to show progress, and timers for contractions. Research will be undertaken in Moray, North East Scotland to increase understanding of the experience of community dwelling women in using apps for PFMT. This study forms part of doctoral research which will also include an explanatory randomised controlled trial and post-trial interviews.

**Aims and objectives:**

The study aims to increase understanding of the motivation and adherence of women in undertaking PFMT. The attitudes and views of health professionals and community dwelling women toward mobile phone apps for PFMT will be investigated. Design elements of at least three apps will be reviewed in terms of their potential usefulness in promoting and prompting behaviour change and in the development of exercise habit formation. The acceptability of apps to support PFMT and the different approaches used will also be examined.

**Methods:**

Focus groups will be conducted with health professionals and community dwelling women from Moray, North East Scotland. The health professionals will be asked to review the quality of health information and to consider implications for potential use by patients. The community dwelling women will be asked for their views on usability and acceptability and for their responses to the different elements and approaches of the apps. The groups will include; mothers of young children, perimenopausal women and older women to represent different age groups.

**Results:**

Data from focus groups around attitudes to, and acceptability of, PFMT apps will be presented at the Congress.

**Conclusions:**

Results from this study will increase understanding about motivation for PFMT and the role of apps in encouraging women to exercise to prevent and improve incontinence symptoms and will be used to design future research.

